# Supplementary figures and images for: TINF2 is a haploinsufficient tumor suppressor that limits telomere length
Source: eLife. 2020 Dec 1;9:e61235. doi: 10.7554/eLife.61235 (PMC7707837; doi:10.7554/eLife.61235)

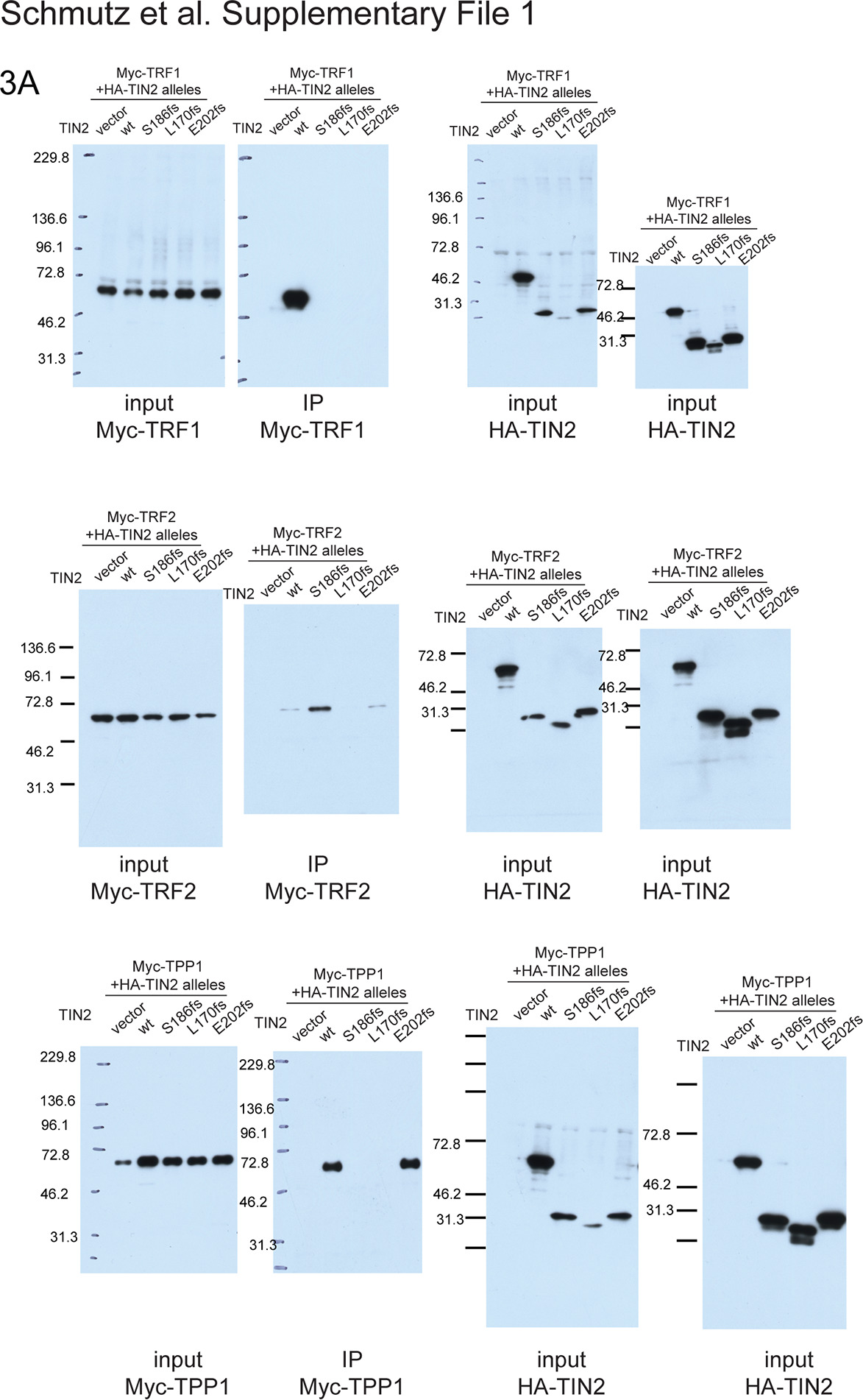

Supplement: Supplementary file 1. [file elife-61235-supp1.jpg]

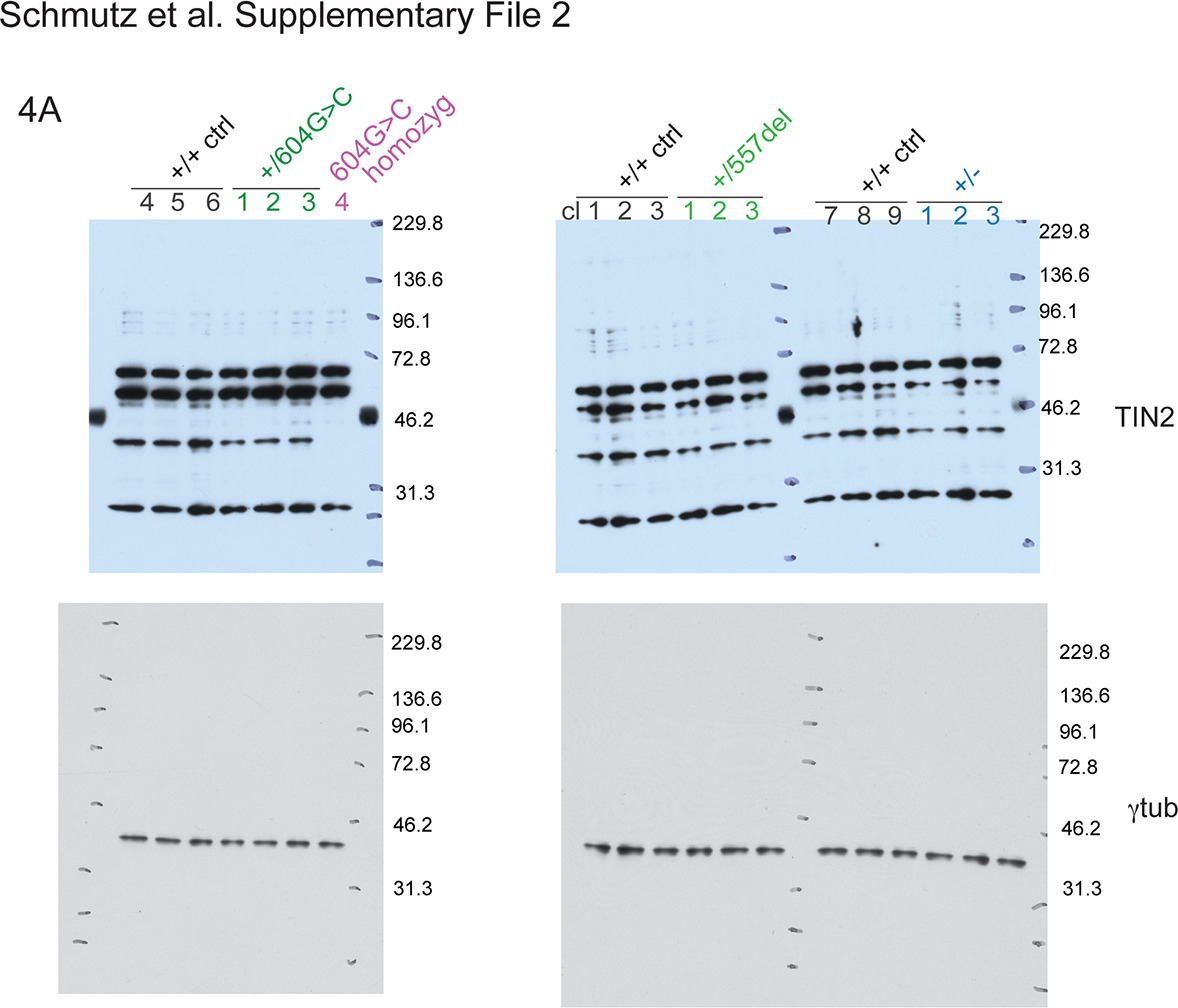

Supplement: Supplementary file 2. [file elife-61235-supp2.jpg]

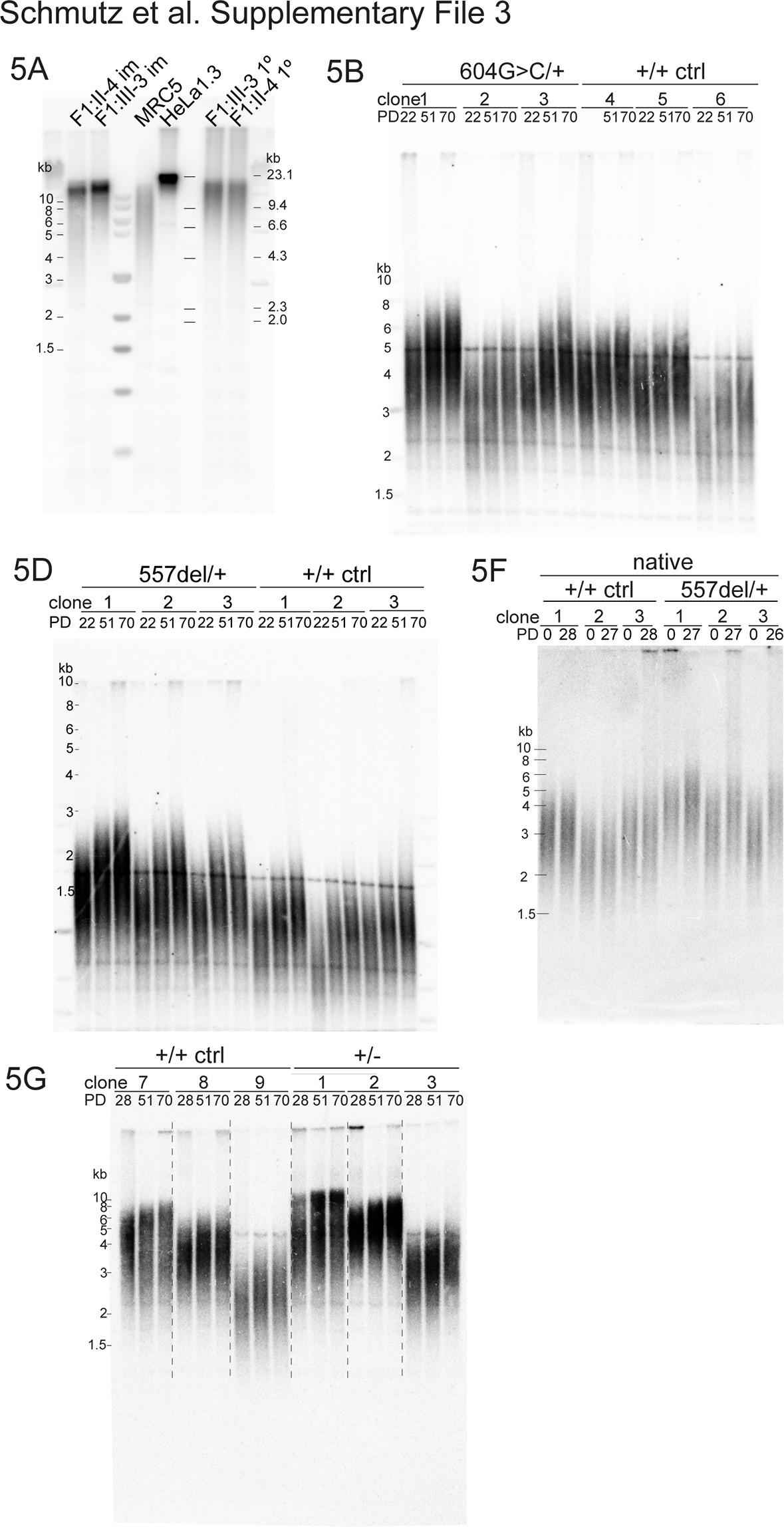

Supplement: Supplementary file 3. [file elife-61235-supp3.jpg]
