# Supplementary material for: TINF2 is a haploinsufficient tumor suppressor that limits telomere length
Source: eLife. 2020 Dec 1;9:e61235. doi: 10.7554/eLife.61235 (PMC7707837; doi:10.7554/eLife.61235)
Supplement: Supplementary file 4. [file elife-61235-supp4.pdf]

Figure	comparison	p value	test	F (DFn, DFd)
3e	vector-wt	<0.0001	unpaired t-test	
	wt-S186fs	<0.0001	unpaired t-test	
	wt-L170sf	<0.0001	unpaired t-test	
	wt-E202fs	0.004	unpaired t-test	
	vector-E202fs	0.1419	unpaired t-test	
4b	"+/+ 1-3 vs +/557del 1-3"	0.023	unpaired t-test	
	"+/+ 4-6 vs +/604G>C 1-3"	0.0129	unpaired t-test	
	"+/+ 7-9 vs +/- 1-3"	0.1645	unpaired t-test	
4d	"+/+ 1-3 vs +/557del 1-3"	0.7561	One way Anova/Tukey	30.57 (6, 572
	"+/+ 4-6 vs +/604G>C 1-3"	0.9996	One way Anova/Tukey	30.57 (6, 572
	"+/+ 8-9 vs +/- 1-3"	0.9997	One way Anova/Tukey	30.57 (6, 572
	"+/+ 1-3 vs 604G>C homozyg"	<0.0001	One way Anova/Tukey	30.57 (6, 572
4f	"+/+ 1-3 vs +/557del 1-3"	>0.9999	One way Anova/Tukey	8.497 (6, 422
	"+/+ 4-6 vs +/604G>C 1-3"	>0.9999	One way Anova/Tukey	8.497 (6, 422
	"+/+ 8-9 vs +/- 1-3"	0.6896	One way Anova/Tukey	8.497 (6, 422
	"+/+ 1-3 vs 604G>C homozyg"	<0.0001	One way Anova/Tukey	8.497 (6, 422
	"+/+ 4-6 vs 604G>C homozyg"	<0.0001	One way Anova/Tukey	8.497 (6, 422
	"+/+ 8-9 vs 604G>C homozyg"	0.0005	One way Anova/Tukey	8.497 (6, 422
4g	"+/+ 1-3 vs +/557del 1-3"	0.9893	One way Anova/Tukey	6.457 (6, 422
	"+/+ 4-6 vs +/604G>C 1-3"	0.3212	One way Anova/Tukey	6.457 (6, 422
	"+/+ 8-9 vs +/- 1-3"	0.9947	One way Anova/Tukey	6.457 (6, 422
	"+/+ 1-3 vs 604G>C homozyg"	<0.0001	One way Anova/Tukey	6.457 (6, 422
	"+/+ 4-6 vs 604G>C homozyg"	<0.0001	One way Anova/Tukey	6.457 (6, 422
	"+/+ 8-9 vs 604G>C homozyg"	<0.0001	One way Anova/Tukey	6.457 (6, 422
5c	"+/+ 4-6 vs +/604G>C 1-3"	0.0482	unpaired t-test	
5e	"+/+ 1-3 vs +/557del 1-3"	0.0007	unpaired t-test	
5h	"+/+ 7-9 vs +/- 1-3"	0.0272	unpaired t-test	
5j	ctrl vs +/-	0.0003	unpaired t-test	

)
)
)
)

)
)
)
)
)
)

)
)
)
)
)
)
